# Supplementary material for: Image classification of forage grasses on Etuoke Banner using edge autoencoder network
Source: PLoS One. 2022 Jun 10;17(6):e0259783. doi: 10.1371/journal.pone.0259783 (PMC9187126; doi:10.1371/journal.pone.0259783)
Supplement: Attachment — Submitted filename: Response to reviewers.docx [file pone.0259783.s001.docx]

Dear Editors and Reviewers:

Thank you very much for your constructive comments and valuable suggestions. We have amended the manuscript accordingly. The changes and corrections are marked in yellow in the manuscript. This document summarizes our revisions and responses in the following. Please have another look to see if they have met your requirements. Thank you for your time!

**Comments from Editors:**

1. The Editor’s comment:

1. Please ensure that your manuscript meets PLOS ONE's style requirements, including those for file naming. The PLOS ONE style templates can be found at

https://journals.plos.org/plosone/s/file?id=wjVg/PLOSOne_formatting_sample_main_body.pdf and [https://journals.plos.org/plosone/s/file?id=ba62/PLOSOne_formatting_sample_title_authors_ affiliations.pdf](https://journals.plos.org/plosone/s/file?id=ba62/PLOSOne_formatting_sample_title_authors_%20affiliations.pdf)

The authors’ answer:

I have revised some of the formatting and I will do my best to make my manuscript conform to PLOS ONE's style.

2. The Editor’s comment:

2. In your Methods section, please provide additional information regarding the permits you obtained for the work. Please ensure you have included the full name of the authority that approved the field site access and, if no permits were required, a brief statement explaining why.

The authors’ answer:

Inner Mongolia University has conducted scientific research with Yiwei White Velvet Goat Co., Ltd. for more than ten years, and opened a test base in the ranch of Yiwei White Velvet Goat Co., Ltd. The data collected are licensed by the company, there is no conflict of interest, if necessary, I can ask Yiwei White Velvet Goat Co., Ltd. to issue a certificate.

3. The Editor’s comment:

In your Methods section, please provide additional location information, including geographic coordinates for the data set if available.

The authors’ answer:

Thank you very much for the Editor’s suggestion. I have added this information in this paper.

The forage grasses images used in the experiment were collected from Erdos City, Etuoke

Banner, Inner Mongolia Yiwei White Cashmere Goat Co, Ltd. and No.1 Branch factory. The longitude and latitude of data acquisition are 39°10′25.8420″N and 107°54′35.8380″E.

4. The Editor’s comment:

We note that Figure 2 in your submission contain map images which may be copyrighted. All PLOS content is published under the Creative Commons Attribution License (CC BY 4.0), which means that the manuscript, images, and Supporting Information files will be freely available online, and any third party is permitted to access, download, copy, distribute, and use these materials in any way, even commercially, with proper attribution. For these reasons, we cannot publish previously copyrighted maps or satellite images created using proprietary data, such as Google software (Google Maps, Street View, and Earth). For more information, see our copyright guidelines: http://journals.plos.org/plosone/s/licenses-and-copyright.

The authors’ answer:

Thank you very much for your suggestion, I have removed the figures from your submission.

**Comments from Reviewer: 1**

1. The reviewer’s comment:

In the abstract, the background knowledge on the problem addressed need to be added.

The authors’ answer:

Thank you very much for the reviewer's suggestion. We have added the problem addressed in the abstract.

Automatically identifying the forage is the basis of intelligent fine breeding of cattle and sheep. Also, it is a key step to study the relationship between the type and quantity of forage collected by cattle and sheep and their own growth, cashmere fineness, milk quality, meat quality and flavor, and so on. The traditional method mainly relies on manual observation, which is time-consuming, laborious and inaccurate, and affects the normal grazing behavior of livestock. In this paper, the optimized convolution neural network: edge autoencoder network(E-A-Net) algorithm is proposed to accurately identify the forage species, which provides the basis for ecological workers to carry out grassland evaluation, grassland management and precision feeding.

We constructed the first forage grass dataset of Etuoke Banner. The forage dataset contains 3889 images in 22 categories. The data is preprocessed and balanced by random cutout data enhancement, the background is removed by image segmentation based on a threshold value.E-A-Net improves the accuracy of herbage recognition in complex background. In order to avoid the phenomenon of richer edge information disappearing in the process of multiple convolutions, a Sobel operator is added in the E-A-Net to extract the edge information of forage grasses. These information is integrated with the features extracted from the backbone network in multi-scale. To avoid the localization of the whole information during the convolution process or alleviate the problem of the whole information disappearance, the pre-training autoencoder network is added to form a hard attention mechanism, which fuses the abstracted overall features of forage grasses with the features extracted from the backbone Convolutional Neural Networks (CNN). Compared with the basic convolutional neural network, alleviates the problem of edge information disappearing and overall feature disappearing with the deepening of network depth. Additionally, compared with the benchmark VGG16, ResNet-50 and EfficientNetB0, the f1-score of the proposed method is improved by 1.6%, 2.8% and 3.7% respectively.

2. The reviewer’s comment:

In the abstract, the wide range of applications and its possible solutions need to be added.

The authors’ answer:

Thank you very much for the reviewer's suggestion. I have added to the abstract a condensation of the applications of pasture identification as well as the problems solved.

Automatically identifying the forage is the basis of intelligent fine breeding of cattle and sheep. Also, it is a key step to study the relationship between the type and quantity of forage collected by cattle and sheep and their own growth, cashmere fineness, milk quality, meat quality and flavor, and so on. The traditional method mainly relies on manual observation, which is time-consuming, laborious and inaccurate, and affects the normal grazing behavior of livestock. In this paper, the optimized convolution neural network algorithm is adopted to accurately identify the forage species, which provides the basis for ecological workers to carry out grassland evaluation, grassland management and precision feeding.

3. The reviewer’s comment:

In the abstract, the problem addressed need to be justified with more details.

The authors’ answer:

Thank you very much for the reviewer's suggestion. I have added some details in the second half of the abstract.

In traditional machine learning algorithms for grass recognition, to reduce the data dimension and the amount of parameters, people only utilize the edge feature information to solve the grass recognition problem, which shows that the edge information of grasses is an important basis for grass recognition, Therefore, we adopt Sobel operator to extract the edge information of forage in four directions, and then fuse these features of multiple scales into the main feature extraction network. This is mainly to solve the problem that the edge information of forage disappears with the deepening of the network.

Autoencoder network is widely used in image denoising, image inpainting, image super-resolution enhancement and other fields. Its network structure mainly consists of two parts: encoding network and decoding one. The image after encoding network is abstracted into important semantic information, which is then converted into original information through decoding network. The important semantic information of the middle layer contains the overall features of the original image. Therefore, we utilize the features of the middle layer extracted from the Autoencoder network, and fuse them with the features obtained from the main feature extraction network and the edge extraction network. This is mainly to play a role in preventing the important overall features from disappearing in the deeper convolution process.

4. The reviewer’s comment:

In the Introduction section, the drawbacks of each conventional technique should be described clearly.

The authors’ answer:

Thank you very much for your constructive comments. For this problem, some modifications are made. We add a new chapter, focusing on convolution neural network technology and traditional machine learning technology for grass recognition, and point out its advantages and disadvantages, as well as the direction of improvement.

Traditional machine learning algorithms and CNN have been widely used in computer vision, and achieved good results. We sorted out the current popular machine learning and deep learning methods about forage recognition. Since most of the grass data sets are built for themselves, the experimental results of different algorithms for different data sets can not be compared objectively, so the grass recognition technology will be elaborated according to the time line.

Deep convolutional neural networks.

Vo Hoang Trong et al. have proposed a yielding multi-fold training (YMufT) strategy to train a DNN model on an imbalanced dataset. This strategy reduces the bias in training through a min-class-max-bound procedure (MCMB), which divides samples in the training set into multiple folds. The model is consecutively trained on each one of these folds. They carried out a series of experiments on several datasets, and the results show that the proposed algorithm has high accuracy.

Shahbaz KhanI et al. have proposed an optimized semi-supervised learning approach, offering a semi-supervised generative adversarial network for crops and weeds classification at early growth stage. In this method, only a few labeled data can be used to complete the weed classification by using the Generative Adversarial Networks (GAN). The proposed system is evaluated extensively on the Red Green Blue (RGB) images obtained by a quadcopter in two different croplands (pea and strawberry). This method achieves 90% accuracy when 80% of training data are not labeled. It has a very good effect for the future development of agricultural intelligence, but the GAN depends on a large number of data sets to generate realistic training data, and for the two classification, 90% accuracy is not high enough.

Yanlei Xu et al. have proposed a weed recognition method using depthwise separable convolutional neural network based on deep transfer learning. In this method, convolution neural network is used to extract grass features, global pooling is used to replace full connection to extract feature vector, and xgboost is used as classifier instead of full connection layer to complete classification. Although this method achieves high accuracy, xgboost belongs to the older tree algorithm, and the newer machine learning classification algorithms may obtain higher accuracy. Besides, the data set is an unbalanced data set, which will affect the generalization ability of the model.

Xue Yan et al. have employed traditional machine learning algorithms to extract edge, color and texture for classification, and compared with DCNN algorithms. Results have demonstrate DCNN algorithms obtain the highest accuracy. It can be concluded that the deep learning algorithms have stronger feature extraction ability than traditional algorithms. In addition, the model structure is relatively simple. It can achieve higher accuracy in the recently proposed image classification algorithms. Kun Hu et al have proposed a novel graph-based deep learning architecture, namely Graph Weeds Net(GWN), which aims to recognize multiple types of weeds from conventional RGB images collected from complex rangelands. The algorithm combines graph convolution neural network, LSTM algorithm and image classification algorithm to complete the classification of eight kinds of weeds. Although the final classification effect is improved, the complexity of the network model is also increased.

Vo Hoang Trong et al. have developed a novel classification approach via a voting method by using the late fusion of multimodal Deep Neural Networks (DNNs). The score vector used for voting is calculated by Bayesian conditional probability method or by determining the priority, so that the score vector obtained from the classification model can get more weight in the final classification. This method can improve the accuracy of the network model, however, the model’s complexity will increase exponentially with the increase of the number of model parameters.

Adnan Farooq et al. have proposed a patch-based classification approach. They evaluated and compared convolutional neural network (CNN) and histogram of oriented gradients (HoG) methods, and concluded that CNN has better feature extraction ability for the image and the accuracy of CNN is greatly affected by the size of the input image.

Alex Olsen contributes the first large, public, multiclass image dataset of weed species from the Australian rangelands, allowing for the development of robust classification methods to make robotic weed control viable. The DeepWeeds dataset consists of 17,509 labeled images of eight nationally significant weed species native to eight locations across northern Australia. They used the benchmark deep learning algorithm to train and identify the data set, and finally achieved 95.7% accuracy.

Mads Dyrmann et al. have presented a method that is capable of recognizing plant species in colour images by using a convolutional neural network. By changing light conditions and soil types to increase the complexity of the dataset, 22 kinds of weeds and crops, a total of 10413 images were taken. And 86.2% accuracy is achieved on the network designed by themselves, but the processing time of a single plant is 27 ms, which is not strong for the real-time detection of weeds.

Traditional machine learning.

Shanwen Zhang et al. proposes a LWMDP based weed recognition method. In the method, Grabcut is firstly used to remove the most background, FMC is utilized to fast segment the weed image, and then LWMDP is employed to reduce the dimension of the segmented weed image, finally SVM is adopted to recognize weed species.This method is not only effective, but also eliminates the interference of background information. However, due to the binarization operation, dimension reduction will lose a lot of details of grasses uncontrollably.

Adel Bakhshipou et al. adopted Fourier descriptors and moment invariant features to extract different shape characteristics of weeds. After fusing these several features, they utilized SVM as classifier and obtained the highest accuracy, overall accuracy of 95.00%. This method mainly relies on the shape features of weeds, but ignores the details of weeds, such as texture, color difference and so on. If the useful feature information is ignored, the final classification effect will be reduced.

David Hall et al. used deep convolutional neural network (ConvNet) features and introduced a range of condition variations to explore the robustness of these features, including: translation, scaling, rotation, shading and occlusion. Evaluations on the Flavia dataset demonstrate that in ideal imaging conditions, combining traditional and ConvNet features yields state-of-the-art performance with an average accuracy of 97.3%±0.6%.

Luís A.M. Pereira et al. established the image data set of aquatic weed leaves, applied five kinds of shape analysis and six kinds of supervised machine learning technology for comparative analysis, and finally achieved 96.41% accuracy on the data set by using the Optimum-path Forestsuan algorithm. These two methods have limited effect on the recognition of leaves in the natural environment because they use the leaves collected separately.

Asnor Juraiza Ishak et al used the combination of Gabor wavelet (GW) and gradient field distribution (GFD) technology to extract a new set of feature vectors for weed classification. The overall classification accuracy is 94% using this technique, while 84% using only the features of GW algorithm. Compared with GW algorithm, GFD algorithm improves the objective accuracy, but it can identify fewer kinds of objects, so it may not be suitable for multiple object data sets.

From the above grass classification dataset and algorithms, it can be seen that for the complex weed dataset, the depth convolution neural network algorithm with stronger learning ability is generally used, while for the relatively simple weed data sets, the machine learning algorithm is typically used. Generally, the machine learning algorithm has simple models and is suitable for processing simple low dimensional data, For image data, it is generally necessary to reduce the dimension by binarization, and then use machine learning algorithm for classification. However, in the field, the growth environment of weeds is very complex, so using deep convolution neural network to classify them is a more reliable strategy. For deep convolutional neural networks, the main problem is that with the deepening of the network, the shallow features will disappear inevitably. ResNet, a network with residual connections, alleviates this problem. But inevitably, some features that are helpful to classification will disappear in the process of convolution. To alleviate this problem, we propose an edge autoencoder network. The extracted edge information and overall feature information are enhanced and input to the basic convolutional neural network to improve the accuracy of the network model.

5. The reviewer’s comment:

Introduction section can be extended to add the issues in the context of the existing work.

The authors’ answer:

Thank you very much for your constructive comments. In the related work chapters, we analyze each convolution technology, and elaborate the existing problems.

6. The reviewer’s comment:

Literature review techniques have to be strengthened by including the issues in the current system and how the author proposes to overcome the same.

The authors’ answer:

Thank you very much for your constructive comments.

In this paper, in order to solve the influence of grass data imbalance on the training network model, we adopt cutout enhancement algorithm to balance the data. Additionally, to decrease the influence of image background, we employ a image segmentation algorithm based on threshold to remove the background information. For the purpose of making better use of biomorphic information of grazing images , hard attention mechanism is utilized to improve classification efficiency in CNN. Furthermore, a Sobel operator is used to extract edge features of grazing images and a pre-training autoencoder network is used to extract abstract global features. Edge extraction network mainly prevents the disappearance of edge information in the deeper convolution. Afterwards, the extracted grazing features are combined with the basic CNN algorithm, namely, edge autoencoder network(E-A-Net). The algorithm utilizes multi-scale forage edge features to prevent the low-level characteristics of forage edge from disappearing with the depth of the network model. The autoencoder network effectively extracts the abstract global features, which plays a role in preventing the CNN from focusing only on the significant features of forage and ignoring the overall features.

7. The reviewer’s comment:

What is the motivation of the proposed work?

The authors’ answer:

Thank you very much for your constructive comments.

In this paper, we mainly design two network algorithms to extract different edge features and overall features. They are used to assist the main feature extraction network to classify. And we also propose a new new algorithm, named E-A-Net.

1: Dataset construction: in the process of data collection, we have completed the collection of forage data completely in different periods under natural conditions. The herbage data collected under natural conditions have more complex illumination and shadow interference than the grasses captured in laboratory conditions, which will increase the experimental difficulty. Yet it is helpful to solve the forage identification in practical application.

2: cutout data enhancement:

There are some differences in the distribution and quantity of different forage data sets. If the quantity is too small, in the process of training network, the algorithm will have higher sensitivity to a small quantity of forage, which is not friendly to the final trained network model. Now, the data enhancement algorithms for data enhancement including Mixup, Cutout, CutMix, Mosaic, have demonstrated that they are very helpful for the learning of the final network model. Therefore, we utilize the cutout data enhancement algorithm to achieve the purpose of increasing the amount of data and balancing data.

3:Image segmentation: experiments show that the soil environment of different grasses will be different in the growth process. If image segmentation operation is not carried out, the soil characteristics will also be used as an important basis for forage recognition, and this kind of forage may obtain high accuracy. However, it is not based on the characteristic information about the forage learned by the network model, but based on its unique soil characteristics. As can be seen from the figure.

4:The edge extraction network is mainly used to extract the rich edge information of the grass. When using traditional machine learning algorithms to classify, the image of the grass or weeds is usually binarized firstly, then the edge is extracted. The purpose of binarization and edge extraction is to reduce the data dimension, because these traditional algorithms are generally not strong in high dimensional data, and they can only use the main features efficiently. In addition, the binary edge extraction operation will cause the lose of texture and color features of the grass image completely, which is not beneficial for grass classification. The data sets used in traditional algorithms are usually very simple, only relying on the image features of grass leaves or the image features with removed background. Obviously, it is difficult for the future practical application.

5:For deep learning algorithms, they do not depend on too much preprocessing, binarization and other operations in the learning process of forage characteristics, and will automatically extract important feature information. However, with the deepening of network model, the edge information of forage will be more abstract, and finally some edge information will disappear. For this reason, we design a convolutional neural network using Sobel operator to extract edge information. It has more shallower structure, and can retain the edge information to the greatest extent. In our experiments, we can see that the edge of the forage image has a higher thermal value, which proves that edge information plays an important role. It can be seen that this network can effectively help improve the classification effect.

6:Autoencoder network is widely used in image restoration, image denoising, obtaining high-dimensional semantic information, and so on. In image classification fields, some papers adopt autoencoder network as an attention mechanism to improve the accuracy of the network model. Inspired by them, we also employ autoencoder. The difference is that they obtain the parameters of autoencoder in the process of continuous training, while we conduct pre-training operation on autoencoder network, and combine the features of the middle layer of autoencoder network with the edge extraction network and the main feature extraction network. The main function of autoencoder network is to prevent the overall features of image from disappearing with the deepening of network.

8. The reviewer’s comment:

Research gaps, objectives of the proposed work should be clearly justified.

The authors’ answer:

Thank you very much for your constructive comments.

There are three main innovations in this paper

1: This is the first time to study the forage grass dataset of Etuoke Banner.

2: We propose an edge feature extraction network, which utilizes the rich edge information of herbage to assist the main feature extraction network to classify.

3: The pre-trained autoencoder network is used to assist the main feature extraction network to classify.

Our work can be divided into five parts

1:constructing dataset.

Since it is the first time to analyze and study the forage grass in Etuoke Banner, we need to build a complete dataset to support the completion of our experiment.

2: balancing data.

Due to the number of different kinds of grasses being quite different, to alleviate the impact of a small number of grasses on the performance of network model, we carry out the data enhancement algorithm to increase their numbers, so as to achieve the purpose of data balance and acquire a robust network model.

3：Image segmentation: the soil environment of forage may be different and if we do not carry out the segmentation operation, the soil characteristics will be treated as a kind of important feature for recognition. It is possible that this kind of forage will acquire a high accuracy, but this is not based on the feature information learned by network, but its unique soil characteristics. Therefore, we conduct the image segmentation operation to alleviate the background interference for classification.

4：edge feature extraction network，

From the visual feature map of the edge extraction network in Fig 10, it is observed that the edge extraction network extracts and retains the grass edge features very well, and the pixels of the edge part have higher values. The class activation feature map shows that the edge information extracted by the edge extraction network plays an import role in the final E-A-Net classification process.

5：autoencoder network：

From Fig 11(b), it can be seen that most of the main characteristics of forage grasses are retained by the autoencoder network. The stitching of the channels with the backbone feature extraction network can make up for the deficiency that most neural networks have blank feature maps on the feature channels of the deep convolutional layer due to the deepening of the network. From Fig 11 (c) (d), it is concluded that the main network model with an edge extraction network is more widely involved in the final classification decision.

9. The reviewer’s comment:

The authors should consider more recent research done in the field of their study (especially in the years 2018 and 2020 onwards)

The authors’ answer:

Thank you very much for your constructive comments. I have added the following references.

6. Gadekallu TR, Rajput DS, Reddy M, Lakshmanna K, Bhattacharya S, Singh S, et al. A novel PCA–whale optimization-based deep neural network model for classification of tomato plant diseases using GPU. Journal of Real-Time Image Processing. 2020; p. 1–14.

26. Gadekallu T, Alazab M, Kaluri R, Reddy P, Bhattacharya S, Lakshman K, et al. Hand gesture classification using a novel CNN-crow search algorithm. Complex & Intelligent Systems. 2021;doi:10.1007/s40747-021-00324-x.

27. Vasan D, Alazab M, Wassan S, Safaei B, Zheng Q. Image-Based Malware Classification using Ensemble of CNN Architectures (IMCEC). Computers & Security. 2020;92:101748. doi:10.1016/j.cose.2020.101748.

28. Bhattacharya S, Reddy Maddikunta PK, Pham QV, Gadekallu TR, Krishnan S SR, Chowdhary CL, et al. Deep learning and medical image processing for coronavirus (COVID-19) pandemic: A survey. Sustainable Cities and Society. 2021;65:102589. doi: https: //doi.org /10.1016/j.scs. 2020. 102589

29. Khan S, Tufail M, Khan M, Ahmad Z, Iqbal J, Alam M. A novel semi-supervised framework for UAV based crop/weed classification. PLOS ONE. 2021;16:e0251008. doi:10.1371/journal.pone.0251008.

30. Yanlei Xu BZYJSKYZZG Yuting Zhai. Weed Recognition for Depthwise Separable Network Based on Transfer Learning. Intelligent Automation & Soft Computing. 2021;27(3):669–682. doi:10.32604/iasc.2021.015225.

31. Yanlei Xu BZYJSKYZZG Yuting Zhai. Weed Recognition for Depthwise Separable Network Based on Transfer Learning. Intelligent Automation & Soft Computing. 2021;27(3):669–682. doi:10.32604/iasc.2021.015225.

32. Yan X, Deng X, Jin J. Classification of weed species in the paddy field with DCNN-Learned features; 2020. p. 336–340.

33. Hu K, Coleman G, Zeng S, Wang Z, Walsh M. Graph weeds net: A graph-based deep learning method for weed recognition. Computers and Electronics in Agriculture. 2020;174:105520. doi:https://doi.org/10.1016/j.compag.2020.105520.

34. Hoang Trong V, Gwang-hyun Y, Thanh Vu D, Jin-young K. Late fusion of multimodal deep neural networks for weeds classification. Computers and Electronics in Agriculture. 2020;175:105506. doi:https://doi.org/10.1016/j.compag.2020.105506.

35. Farooq A, Hu J, Jia X. Analysis of Spectral Bands and Spatial Resolutions for Weed Classification Via Deep Convolutional Neural Network. IEEE Geoscience and Remote Sensing Letters. 2019;16(2):183–187. doi:10.1109/LGRS.2018.2869879.

36. Olsen A, Konovalov DA, Philippa B, Ridd P, Wood JC, Johns J, et al. DeepWeeds A Multiclass Weed Species Image Dataset for Deep Learning. Scientific Reports. 2019;9(1). doi:10.1038s41598-018-38343-3.

37. Dyrmann M, Karstoft H, Midtiby HS. Plant species classification using deep convolutional neural network. Biosystems Engineering. 2016;151:72–80. doi:https://doi.org/10.1016/j.biosystemseng.2016.08.024.

38. Zhang S, Guo J, Wang Z. Combing K-means Clustering and Local Weighted Maximum Discriminant Projections for Weed Species Recognition. Frontiers in Computer Science. 2019;1:4. doi:10.3389/fcomp.2019.00004.

39. Bakhshipour A, Jafari A. Evaluation of support vector machine and artificial neural networks in weed detection using shape features. Computers and Electronics in Agriculture. 2018;145:153–160. doi:https://doi.org/10.1016/j.compag.2017.12.032.

40. Hall D, McCool C, Dayoub F, Sunderhauf N, Upcroft B. Evaluation of Features for Leaf Classification in Challenging Conditions. In: 2015 IEEE Winter Conference on Applications of Computer Vision; 2015. p. 797–804.

41. Pereira LAM, Nakamura RYM, de Souza GFS, Martins D, Papa JP. Aquatic weed automatic classification using machine learning techniques. Computers and Electronics in Agriculture. 2012;87:56–63. doi:https://doi.org/10.1016/j.compag.2012.05.015.

42. Ishak AJ, Hussain A, Mustafa MM. Weed image classification using Gabor wavelet and gradient field distribution. Computers and Electronics in Agriculture. 2009;66(1):53–61. doi:https://doi.org/10.1016/j.compag.2008.12.003.

10. The reviewer’s comment:

An error and statistical analysis of data should be performed.

The authors’ answer:

Thank you very much for your constructive comments.

As can be seen from Fig 9, E-A-VGG16 and E-A-ResNet50 are faster than VGG16 and ResNet50 in learning speed. A-EfficientNetB0 network trained on a forage grass dataset is more accurate both in the training set and in the validation set than in the original classification network, and the result curve is more stable in the validation set.

The above 12 curves can more accurately show the accuracy changes of different models during the training process. We can see that after 100 rounds the accuracy waveform of the network model tends to be smooth and rises slowly. Therefore, we select the last 100 accuracy values and 7 indicators to study the performance of the algorithm.

|  | Count | Mean | std | Median | MIN | Max | Difference |
| --- | --- | --- | --- | --- | --- | --- | --- |
| VGG16_Train | 100 | 0.981587 | 0.005450 | 0.982298 | 0.965738 | 0.992691 | 0.011104 |
| VGG16_Valid | 100 | 0.938200 | 0.021919 | 0.941204 | 0.817593 | 0.970370 | 0.032170 |
| E-A-VGG16_Train | 100 | 0.995430 | 0.002451 | 0.996110 | 0.984668 | 0.998856 | 0.003426 |
| E-A-VGG16_Valid | 100 | 0.962222 | 0.006415 | 0.962963 | 0.943519 | 0.975000 | 0.012778 |
| ResNet50_Train | 100 | 0.982360 | 0.005039 | 0.983212 | 0.969849 | 0.993604 | 0.011244 |
| ResNet50_Valid | 100 | 0.951850 | 0.018886 | 0.956481 | 0.875000 | 0.983333 | 0.031483 |
| E-A- ResNet50_Train | 100 | 0.995386 | 0.001631 | 0.996574 | 0.991092 | 0.998401 | 0.003015 |
| E-A-ResNet50_Valid | 100 | 0.971361 | 0.003905 | 0.971296 | 0.961111 | 0.981481 | 0.010119 |
| EfficientNetB0_Train | 100 | 0.860690 | 0.038122 | 0.870375 | 0.775468 | 0.914344 | 0.053654 |
| EfficientNetB0_ Valid | 100 | 0.866204 | 0.045947 | 0.870833 | 0.719444 | 0.955556 | 0.089352 |
| E-A-EfficientNetB0_Train | 100 | 0.940998 | 0.013105 | 0.945523 | 0.897899 | 0.958429 | 0.017431 |
| E-A-EfficientNetB0_Valid | 100 | 0.947398 | 0.010777 | 0.950463 | 0.911111 | 0.962037 | 0.014639 |

In training set, E-A-VGG16 has the highest accuracy compared with other algorithms, 0.998856. EfficientNetB0 has the worst performance, 0.914344. Compared with other algorithms, E-A-ResNet5 has the minimum standard deviation at 0.001631, while EfficientNetB0 has the maximum standard deviation at 0.038122. Therefore, it can be concluded that E-A- ResNet50 has a gentle learning and small fluctuation in the training set. This conclusion can also be verified by the waveform fluctuation of E-A- ResNet50 compared with other algorithms. Meanwhile, according to the maximum and median, we can estimate the accuracy improvement of the network model after 150 rounds. We can draw the conclusion that the accuracy improvement of EfficientNetB0 is up to 0.053654, and the improvement of E-A-ResNet50 is at least 0.003015. This shows E-A-ResNet50 has strong learning ability.

In validation set, E-A-ResNet50 has the highest accuracy compared with other algorithms, 0.981481. EfficientNetB0 has the worst performance, $0.955556$. Compared with other algorithms, E-A-ResNet50 has the minimum standard deviation with $0.003905$ while EfficientNetB0 has the maximum standard deviation with $0.045947$. It can be seen that E-A- ResNet50 has stable effect and high accuracy in the validation set. According to median and maximum, the accuracy improvement of network model after 150 rounds can be estimated. The accuracy of E-A-ResNet50 is 0.010119 while that of EfficientNetB0 is the highest, $0.089352$. In short, we can get a consistent conclusion: E-A-ResNet50 has a strong learning ability, and in the first 150 rounds of training, it has basically completed the parameter learning.

Meanwhile, we also find a phenomenon which is inconsistent with the other two models. For EfficientNetB0 and E-A-EfficientNetB0, their accuracy in the validation set is generally higher than that in the training set, which proves that the algorithm has good generalization ability. Compared with the other four algorithm, these four algorithms’ accuracy is generally higher in the training set than that in the validation set, which indicates that these four algorithms have slight overfitting. Therefore, we choose the most accurate weight in the validation set to make the final evaluation of the above six models on the test set.

F1 − score is used as the final standard to evaluate the model performance. E-A-VGG16, E-A-ResNet50 and E-A-EfficientNetB0 are increased by $1.6\%$, $2.8\%$ and $3.7\%$ respectively than VGG16, and EfficientNetB0. E-A-VGG16, E-A-Resnet50 and E-A-EfficientnetB0 have more, $3.4\times{10}^{6}$、$16.1 \times{10}^{6}$ 、 $12.1 \times{10}^{5}$ parameters than VGG16, ReNet-50 and EfficientNetB0 respectively. In terms of the processing speed for a single image, E-A-VGG16, E-A-ResNet50 and E-A-EfficientNetB0 save 0.0045s, 0.00528s, 0.0028s respectively than VGG16, ReNet-50 and EfficientNetB0. Experimental results show that although there are more parameters in these three improved networks, the accuracy is greatly improved, especially in the E-A- EfficientNetB0. Only $12.1 \times{10}^{5}$ parameters and 0.0028s of single image operation time are added, but the test accuracy is improved by 3.7%. Therefore, the fusion of the features extracted from the edge extraction network and the features extracted from the autoencoder network into the main feature extraction network can effectively improve the accuracy of the network model and the generalization ability .

From the above experimental results, it can be concluded that although E-A-EfficientNetB0 has lower accuracy in the training set and verification set than that of E-A-VGG16, E-A-ResNet50, VGG16 and ResNet50, in test set it has the same effect as E-A-VGG16, and is higher than the other four algorithms in each index. Compared with E-A-VGG16, E-A-EfficientNetB0 has fewer parameters and shorter processing time for a single image. Therefore, E-A-EfficientNetB0 has better performance on forage data sets, which proves the effectiveness of our proposed edge autoencoder network.

11. The reviewer’s comment:

The conclusion should state scope for future work.

The authors’ answer:

Forage recognition technology can be used in intelligent animal husbandry projects in the future. Therefore, there are still many aspects to be improved in this experiment, and there is still much work to be done in the future. I will list some areas that can be improved in the future.

1: Expansion of dataset: the leaf texture, shape and color of herbage are not exactly the same even for the same kind of herbage. Besides, the color of herbage in May is light green, that in August is dark green, and that in October is a little yellow. Therefore, in order to obtain a more robust herbage recognition model, it is an important, lasting and time-consuming work to expand the forage dataset.

2: Model simplification: our algorithm improves the accuracy of the model to a certain extent, but also increases model’s parameters. Knowledge distillation can transfer knowledge from one network to another. These two networks can be isomorphic or heterogeneous. It can be used to transform a network from a large network to a small network, and keep the performance close to that of a large network. In the future, we will design a simplified network model so as to increase the reasoning speed of the network model on the premise of ensuring the accuracy.

3: Production of wearable devices: for the purpose of accurately counting the amount and species of forage, we will make a wearable forage recognition device in the future.

According to the sound sensor, we can use the chewing signal of cattle and sheep to control the camera. When the chewing signal is found, we can turn on the camera to obtain the species of forage. The chewing signal is used to count the number and species of forage.

12. The reviewer’s comment:

Discuss the future plans with respect to the research state of progress and its limitations.

The authors’ answer:

Thank you very much for your constructive comments. In the eleventh reply, I have written out the problems of the system and the future direction of work, please criticize and correct me

**Comments from Reviewer: 2**

1.The reviewer’s comment:

The limitations of our work include:

The authors’ answer:

Since edge extraction network and autoencoder network are added into the basic feature extraction network, the number of network model parameters is inevitably increased, which will decrease the calculation efficiency. Based on the comparison experiment, it can be seen that E-A-Net reduces the processing speed of images to different degrees. Hence, in the future, we will use knowledge distillation algorithm to construct a student network with simple model structure and less parameters to learn the output of tutor network of complex network. Without reducing the accuracy of network model, we can get a simpler model architecture and deploy it to the development board to verify its experimental performance.

Under natural conditions the forage growth environment is usually complex. In the future, we will make a wearable equipment for cattle and sheep to identify the forage species, and obtain the number of forage by sensor devices, and further calculate the feed intake of each forage. Although we take photos in real natural environment, the robustness of the network model after training will be increased to some extent. However, the growth of several forages under the real natural environment is far more complicated than this, which is also our further research direction.

2.The reviewer’s comment:

List out the main contributions of the current work.
The authors’ answer:

Thank you very much for your constructive comments. For this problem, some modifications are made. We add a new chapter, focusing on convolution neural network technology and traditional machine learning technology for grass recognition, and point out its advantages and disadvantages, as well as the direction of improvement.

Traditional machine learning algorithms and CNN have been widely used in computer vision, and achieved good results. We sorted out the current popular machine learning and deep learning methods about forage recognition. Since most of the grass data sets are built for themselves, the experimental results of different algorithms for different data sets can not be compared objectively, so the grass recognition technology will be elaborated according to the time line.

Deep convolutional neural networks.

Vo Hoang Trong et al. have proposed a yielding multi-fold training (YMufT) strategy to train a DNN model on an imbalanced dataset. This strategy reduces the bias in training through a min-class-max-bound procedure (MCMB), which divides samples in the training set into multiple folds. The model is consecutively trained on each one of these folds. They carried out a series of experiments on several datasets, and the results show that the proposed algorithm has high accuracy.

Shahbaz KhanI et al. have proposed an optimized semi-supervised learning approach, offering a semi-supervised generative adversarial network for crops and weeds classification at early growth stage. In this method, only a few labeled data can be used to complete the weed classification by using the Generative Adversarial Networks (GAN). The proposed system is evaluated extensively on the Red Green Blue (RGB) images obtained by a quadcopter in two different croplands (pea and strawberry). This method achieves 90% accuracy when 80% of training data are not labeled. It has a very good effect for the future development of agricultural intelligence, but the GAN depends on a large number of data sets to generate realistic training data, and for the two classification, 90% accuracy is not high enough.

Yanlei Xu et al. have proposed a weed recognition method using depthwise separable convolutional neural network based on deep transfer learning. In this method, convolution neural network is used to extract grass features, global pooling is used to replace full connection to extract feature vector, and xgboost is used as classifier instead of full connection layer to complete classification. Although this method achieves high accuracy, xgboost belongs to the older tree algorithm, and the newer machine learning classification algorithms may obtain higher accuracy. Besides, the data set is an unbalanced data set, which will affect the generalization ability of the model.

Xue Yan et al have employed traditional machine learning algorithms to extract edge, color and texture for classification, and compared with DCNN algorithms. Results have demonstrate DCNN algorithms obtain the highest accuracy. It can be concluded that the deep learning algorithms have stronger feature extraction ability than traditional algorithms. In addition, the model structure is relatively simple. It can achieve higher accuracy in the recently proposed image classification algorithms. Kun Hu et al. have proposed a novel graph-based deep learning architecture, namely Graph Weeds Net(GWN), which aims to recognize multiple types of weeds from conventional RGB images collected from complex rangelands. The algorithm combines graph convolution neural network, LSTM algorithm and image classification algorithm to complete the classification of eight kinds of weeds. Although the final classification effect is improved, the complexity of the network model is also increased.

Vo Hoang Trong et al have developed a novel classification approach via a voting method by using the late fusion of multimodal Deep Neural Networks (DNNs). The score vector used for voting is calculated by Bayesian conditional probability method or by determining the priority, so that the score vector obtained from the classification model can get more weight in the final classification. This method can improve the accuracy of the network model, however, the model’s complexity will increase exponentially with the increase of the number of model parameters.

Adnan Farooq et al have proposed a patch-based classification approach. They evaluated and compared convolutional neural network (CNN) and histogram of oriented gradients (HoG) methods, and concluded that CNN has better feature extraction ability for the image and the accuracy of CNN is greatly affected by the size of the input image.

Alex Olsen contributes the first large, public, multiclass image dataset of weed species from the Australian rangelands, allowing for the development of robust classification methods to make robotic weed control viable. The DeepWeeds dataset consists of 17,509 labeled images of eight nationally significant weed species native to eight locations across northern Australia.

They used the benchmark deep learning algorithm to train and identify the data set, and finally achieved 95.7% accuracy. Mads Dyrmann et al. have presented a method that is capable of recognizing plant species in colour images by using a convolutional neural network. By changing light conditions and soil types to increase the complexity of the dataset, 22 kinds of weeds and crops, a total of 10413 images were taken. And 86.2% accuracy is achieved on the network designed by themselves, but the processing time of a single plant is 27 ms, which is not strong for the real-time detection of weeds.

Traditional machine learning.

Shanwen Zhang et al. proposes a LWMDP based weed recognition method. In the method, Grabcut is firstly used to remove the most background, FMC is utilized to fast segment the weed image, and then LWMDP is employed to reduce the dimension of the segmented weed image, finally SVM is adopted to recognize weed species.This method is not only effective, but also eliminates the interference of background information. However, due to the binarization operation, dimension reduction will lose a lot of details of grasses uncontrollably.

Adel Bakhshipou et al. adopted Fourier descriptors and moment invariant features to extract different shape characteristics of weeds. After fusing these several features, they utilized SVM as classifier and obtained the highest accuracy, overall accuracy of 95.00%. This method mainly relies on the shape features of weeds, but ignores the details of weeds, such as texture, color difference and so on. If the useful feature information is ignored, the final classification effect will be reduced.

David Hall et al. used deep convolutional neural network (ConvNet) features and introduced a range of condition variations to explore the robustness of these features, including: translation, scaling, rotation, shading and occlusion. Evaluations on the Flavia dataset demonstrate that in ideal imaging conditions, combining traditional and ConvNet features yields state-of-the-art performance with an average accuracy of 97.3%±0.6%.

Luís A.M. Pereira et al. established the image data set of aquatic weed leaves, applied five kinds of shape analysis and six kinds of supervised machine learning technology for comparative analysis, and finally achieved 96.41% accuracy on the data set by using the Optimum-path Forestsuan algorithm. These two methods have limited effect on the recognition of leaves in the natural environment because they use the leaves collected separately.

Asnor Juraiza Ishak et al used the combination of Gabor wavelet (GW) and gradient field distribution (GFD) technology to extract a new set of feature vectors for weed classification. The overall classification accuracy is 94% using this technique, while 84% using only the features of GW algorithm. Compared with GW algorithm, GFD algorithm improves the objective accuracy, but it can identify fewer kinds of objects, so it may not be suitable for multiple object data sets.

From the above grass classification dataset and algorithms, it can be seen that for the complex weed dataset, the depth convolution neural network algorithm with stronger learning ability is generally used, while for the relatively simple weed data sets, the machine learning algorithm is typically used. Generally, the machine learning algorithm has simple models and is suitable for processing simple low dimensional data, For image data, it is generally necessary to reduce the dimension by binarization, and then use machine learning algorithm for classification. However, in the field, the growth environment of weeds is very complex, so using deep convolution neural network to classify them is a more reliable strategy. For deep convolutional neural networks, the main problem is that with the deepening of the network, the shallow features will disappear inevitably. ResNet, a network with residual connections, alleviates this problem. But inevitably, some features that are helpful to classification will disappear in the process of convolution. To alleviate this problem, we propose an edge autoencoder network. The extracted edge information and overall feature information are enhanced and input to the basic convolutional neural network to improve the accuracy of the network model.

3.The reviewer’s comment:

Some of the recent works on ML/AI such as the following can be discussed in the paper: "Image-Based malware classification using ensemble of CNN architectures (IMCEC), A Novel PCA-Whale Optimization based Deep Neural Network model for Classification of Tomato Plant Diseases using GPU, Deep learning and medical image processing for coronavirus (COVID-19) pandemic: A survey, Hand gesture classification using a novel CNN-crow search algorithm".

The authors’ answer:

I read the above articles in great detail. Their research content is closely related to my work now, and the literature time is relatively new. Additionally, they have high research significance and reference value. Therefore, I quote the above literatures in the introduction of the paper.

6. Gadekallu TR, Rajput DS, Reddy M, Lakshmanna K, Bhattacharya S, Singh S, et al. A novel PCA–whale optimization-based deep neural network model for classification of tomato plant diseases using GPU. Journal of Real-Time Image Processing. 2020; p. 1–14.

26. Gadekallu T, Alazab M, Kaluri R, Reddy P, Bhattacharya S, Lakshman K, et al. Hand gesture classification using a novel CNN-crow search algorithm. Complex & Intelligent Systems. 2021;doi:10.1007/s40747-021-00324-x.

27. Vasan D, Alazab M, Wassan S, Safaei B, Zheng Q. Image-Based Malware Classification using Ensemble of CNN Architectures (IMCEC). Computers & Security. 2020;92:101748. doi:10.1016/j.cose.2020.101748.

28. Bhattacharya S, Reddy Maddikunta PK, Pham QV, Gadekallu TR, Krishnan S SR, Chowdhary CL, et al. Deep learning and medical image processing for coronavirus (COVID-19) pandemic: A survey. Sustainable Cities and Society. 2021;65:102589. doi: https: //doi.org /10.1016/j.scs. 2020. 102589

29. Khan S, Tufail M, Khan M, Ahmad Z, Iqbal J, Alam M. A novel semi-supervised framework for UAV based crop/weed classification. PLOS ONE. 2021;16:e0251008. doi:10.1371/journal.pone.0251008.

30. Yanlei Xu BZYJSKYZZG Yuting Zhai. Weed Recognition for Depthwise Separable Network Based on Transfer Learning. Intelligent Automation & Soft Computing. 2021;27(3):669–682. doi:10.32604/iasc.2021.015225.

31. Yanlei Xu BZYJSKYZZG Yuting Zhai. Weed Recognition for Depthwise Separable Network Based on Transfer Learning. Intelligent Automation & Soft Computing. 2021;27(3):669–682. doi:10.32604/iasc.2021.015225.

32. Yan X, Deng X, Jin J. Classification of weed species in the paddy field with DCNN-Learned features; 2020. p. 336–340.

33. Hu K, Coleman G, Zeng S, Wang Z, Walsh M. Graph weeds net: A graph-based deep learning method for weed recognition. Computers and Electronics in Agriculture. 2020;174:105520. doi:https://doi.org/10.1016/j.compag.2020.105520.

34. Hoang Trong V, Gwang-hyun Y, Thanh Vu D, Jin-young K. Late fusion of multimodal deep neural networks for weeds classification. Computers and Electronics in Agriculture. 2020;175:105506. doi:https://doi.org/10.1016/j.compag.2020.105506.

35. Farooq A, Hu J, Jia X. Analysis of Spectral Bands and Spatial Resolutions for Weed Classification Via Deep Convolutional Neural Network. IEEE Geoscience and Remote Sensing Letters. 2019;16(2):183–187. doi:10.1109/LGRS.2018.2869879.

36. Olsen A, Konovalov DA, Philippa B, Ridd P, Wood JC, Johns J, et al. DeepWeeds A Multiclass Weed Species Image Dataset for Deep Learning. Scientific Reports. 2019;9(1). doi:10.1038s41598-018-38343-3.

37. Dyrmann M, Karstoft H, Midtiby HS. Plant species classification using deep convolutional neural network. Biosystems Engineering. 2016;151:72–80. doi:https://doi.org/10.1016/j.biosystemseng.2016.08.024.

38. Zhang S, Guo J, Wang Z. Combing K-means Clustering and Local Weighted Maximum Discriminant Projections for Weed Species Recognition. Frontiers in Computer Science. 2019;1:4. doi:10.3389/fcomp.2019.00004.

39. Bakhshipour A, Jafari A. Evaluation of support vector machine and artificial neural networks in weed detection using shape features. Computers and Electronics in Agriculture. 2018;145:153–160. doi:https://doi.org/10.1016/j.compag.2017.12.032.

40. Hall D, McCool C, Dayoub F, Sunderhauf N, Upcroft B. Evaluation of Features for Leaf Classification in Challenging Conditions. In: 2015 IEEE Winter Conference on Applications of Computer Vision; 2015. p. 797–804.

41. Pereira LAM, Nakamura RYM, de Souza GFS, Martins D, Papa JP. Aquatic weed automatic classification using machine learning techniques. Computers and Electronics in Agriculture. 2012;87:56–63. doi:https://doi.org/10.1016/j.compag.2012.05.015.

42. Ishak AJ, Hussain A, Mustafa MM. Weed image classification using Gabor wavelet and gradient field distribution. Computers and Electronics in Agriculture. 2009;66(1):53–61. doi:https://doi.org/10.1016/j.compag.2008.12.003.

4.The reviewer’s comment:

Summarize the related works section in the form of a table.

The authors’ answer:

Thank you very much for your constructive comments. I have added a list of related work in chapter

5.The reviewer’s comment:

Compare the current work with recent state-of-the-art.

The authors’ answer:

Thank you very much for your constructive comments.

I am very sorry, because this dataset is a self-collected dataset, no experiments have been done on this dataset, so I can only compare my own algorithm with the base algorithm. We will continue to improve and optimize our algorithm in future experiments to make our algorithm more accurate and faster.

6.The reviewer’s comment:

Present a detailed analysis on results obtained.

The authors’ answer:

Thank you very much for your constructive comments.

As can be seen from Fig 9, E-A-VGG16 and E-A-ResNet50 are faster than VGG16 and ResNet50 in learning speed. A-EfficientNetB0 network trained on a forage grass dataset is more accurate both in the training set and in the validation set than in the original classification network, and the result curve is more stable in the validation set.

The above 12 curves can more accurately show the accuracy changes of different models during the training process. We can see that after 100 rounds the accuracy waveform of the network model tends to be smooth and rises slowly. Therefore, we select the last 100 accuracy values and 7 indicators to study the performance of the algorithm.

|  | Count | Mean | std | Median | MIN | Max | Difference |
| --- | --- | --- | --- | --- | --- | --- | --- |
| VGG16_Train | 100 | 0.981587 | 0.005450 | 0.982298 | 0.965738 | 0.992691 | 0.011104 |
| VGG16_Valid | 100 | 0.938200 | 0.021919 | 0.941204 | 0.817593 | 0.970370 | 0.032170 |
| E-A-VGG16_Train | 100 | 0.995430 | 0.002451 | 0.996110 | 0.984668 | 0.998856 | 0.003426 |
| E-A-VGG16_Valid | 100 | 0.962222 | 0.006415 | 0.962963 | 0.943519 | 0.975000 | 0.012778 |
| ResNet50_Train | 100 | 0.982360 | 0.005039 | 0.983212 | 0.969849 | 0.993604 | 0.011244 |
| ResNet50_Valid | 100 | 0.951850 | 0.018886 | 0.956481 | 0.875000 | 0.983333 | 0.031483 |
| E-A- ResNet50_Train | 100 | 0.995386 | 0.001631 | 0.996574 | 0.991092 | 0.998401 | 0.003015 |
| E-A-ResNet50_Valid | 100 | 0.971361 | 0.003905 | 0.971296 | 0.961111 | 0.981481 | 0.010119 |
| EfficientNetB0_Train | 100 | 0.860690 | 0.038122 | 0.870375 | 0.775468 | 0.914344 | 0.053654 |
| EfficientNetB0_ Valid | 100 | 0.866204 | 0.045947 | 0.870833 | 0.719444 | 0.955556 | 0.089352 |
| E-A-EfficientNetB0_Train | 100 | 0.940998 | 0.013105 | 0.945523 | 0.897899 | 0.958429 | 0.017431 |
| E-A-EfficientNetB0_Valid | 100 | 0.947398 | 0.010777 | 0.950463 | 0.911111 | 0.962037 | 0.014639 |

In training set, E-A-VGG16 has the highest accuracy compared with other algorithms, 0.998856. EfficientNetB0 has the worst performance, 0.914344. Compared with other algorithms, E-A-ResNet5 has the minimum standard deviation at 0.001631, while EfficientNetB0 has the maximum standard deviation at 0.038122. Therefore, it can be concluded that E-A- ResNet50 has a gentle learning and small fluctuation in the training set. This conclusion can also be verified by the waveform fluctuation of E-A- ResNet50 compared with other algorithms. Meanwhile, according to the maximum and median, we can estimate the accuracy improvement of the network model after 150 rounds. We can draw the conclusion that the accuracy improvement of EfficientNetB0 is up to 0.053654, and the improvement of E-A-ResNet50 is at least 0.003015. This shows E-A-ResNet50 has strong learning ability.

In validation set, E-A-ResNet50 has the highest accuracy compared with other algorithms, 0.981481. EfficientNetB0 has the worst performance, 0.955556. Compared with other algorithms, E-A-ResNet50 has the minimum standard deviation with 0.003905 while EfficientNetB0 has the maximum standard deviation with 0.045947. It can be seen that E-A- ResNet50 has stable effect and high accuracy in the validation set. According to median and maximum, the accuracy improvement of network model after 150 rounds can be estimated. The accuracy of E-A-ResNet50 is 0.010119 while that of EfficientNetB0 is the highest, 0.089352. In short, we can get a consistent conclusion: E-A-ResNet50 has a strong learning ability, and in the first 150 rounds of training, it has basically completed the parameter learning.

Meanwhile, we also find a phenomenon which is inconsistent with the other two models. For EfficientNetB0 and E-A-EfficientNetB0, their accuracy in the validation set is generally higher than that in the training set, which proves that the algorithm has good generalization ability. Compared with the other four algorithm, these four algorithms’ accuracy is generally higher in the training set than that in the validation set, which indicates that these four algorithms have slight overfitting. Therefore, we choose the most accurate weight in the validation set to make the final evaluation of the above six models on the test set.

F1 − score is used as the final standard to evaluate the model performance. E-A-VGG16, E-A-ResNet50 and E-A-EfficientNetB0 are increased by $1.6\%$, $2.8\%$ and $3.7\%$ respectively than VGG16, and EfficientNetB0. E-A-VGG16, E-A-Resnet50 and E-A-EfficientnetB0 have more, $3.4\times{10}^{6}$、$16.1 \times{10}^{6}$ 、 $12.1 \times{10}^{5}$ parameters than VGG16, ReNet-50 and EfficientNetB0 respectively. In terms of the processing speed for a single image, E-A-VGG16, E-A-ResNet50 and E-A-EfficientNetB0 save 0.0045s, 0.00528s, 0.0028s respectively than VGG16, ReNet-50 and EfficientNetB0. Experimental results show that although there are more parameters in these three improved networks, the accuracy is greatly improved, especially in the E-A- EfficientNetB0. Only $12.1 \times{10}^{5}$ parameters and 0.0028s of single image operation time are added, but the test accuracy is improved by 3.7%. Therefore, the fusion of the features extracted from the edge extraction network and the features extracted from the autoencoder network into the main feature extraction network can effectively improve the accuracy of the network model and the generalization ability .

From the above experimental results, it can be concluded that although E-A-EfficientNetB0 has lower accuracy in the training set and verification set than that of E-A-VGG16, E-A-ResNet50, VGG16 and ResNet50, in test set it has the same effect as E-A-VGG16, and is higher than the other four algorithms in each index. Compared with E-A-VGG16, E-A-EfficientNetB0 has fewer parameters and shorter processing time for a single image. Therefore, E-A-EfficientNetB0 has better performance on forage data sets, which proves the effectiveness of our proposed edge autoencoder network.

7.The reviewer’s comment:

Discuss about the limitations of the current work in conclusion

The authors’ answer:

Thank you very much for your constructive comments.

Forage recognition technology can be used in intelligent animal husbandry projects in the future. Therefore, there are still many aspects to be improved in this experiment, and there is still much work to be done in the future. I will list some areas that can be improved in the future.

1: Expansion of dataset: the leaf texture, shape and color of herbage are not exactly the same even for the same kind of herbage. Besides, the color of herbage in May is light green, that in August is dark green, and that in October is a little yellow. Therefore, in order to obtain a more robust herbage recognition model, it is an important, lasting and time-consuming work to expand the forage dataset.

2: Model simplification: our algorithm improves the accuracy of the model to a certain extent, but also increases model’s parameters. Knowledge distillation can transfer knowledge from one network to another. These two networks can be isomorphic or heterogeneous. It can be used to transform a network from a large network to a small network, and keep the performance close to that of a large network. In the future, we will design a simplified network model so as to increase the reasoning speed of the network model on the premise of ensuring the accuracy.

3: Production of wearable devices: for the purpose of accurately counting the amount and species of forage, we will make a wearable forage recognition device in the future.

According to the sound sensor, we can use the chewing signal of cattle and sheep to control the camera. When the chewing signal is found, we can turn on the camera to obtain the species of forage. The chewing signal is used to count the number and species of forage.
