# Supplementary material for: Image classification of forage grasses on Etuoke Banner using edge autoencoder network
Source: PLoS One. 2022 Jun 10;17(6):e0259783. doi: 10.1371/journal.pone.0259783 (PMC9187126; doi:10.1371/journal.pone.0259783)
Supplement: Attachment — Submitted filename: Response to reviewers.docx [file pone.0259783.s002.docx]

Dear Editors and Reviewers:

Thank you very much for your constructive comments and valuable suggestions. We have amended the manuscript accordingly. The changes and corrections are marked in yellow in the manuscript. This document summarizes our revisions and responses in the following. Please have another look to see if they have met your requirements. Thank you for your time!

**Comments from Editors:**

1. The Editor’s comment:

1. Please review your reference list to ensure that it is complete and correct. If you have cited papers that have been retracted, please include the rationale for doing so in the manuscript text, or remove these references and replace them with relevant current references. Any changes to the reference list should be mentioned in the rebuttal letter that accompanies your revised manuscript. If you need to cite a retracted article, indicate the article’s retracted status in the References list and also include a citation and full reference for the retraction notice.

The authors’ answer:

Thanks for your constructive suggestions! We have modified the reference format so as to meet the requirements of this journal. The specific modified format is as follows:

1. Rutter S. Review: Grazing preferences in sheep and cattle: Implications for production, the environment and animal welfare. Canadian Journal of Animal Science. 2010;90:285–293.

2. Rombach M, M¨unger A, Niederhauser J, S¨udekum KH, Schori F. Evaluation and validation of an automatic jaw movement recorder (RumiWatch) for ingestive andrumination behaviors of dairy cows during grazing and supplementation. Journal of Dairy Science. 2018;101(3):2463 – 2475.

3. Moln´ar Z. “I See the Grass Through the Mouths of My Animals” – Folk Indicators of Pasture Plants Used by Traditional Steppe Herders. Journal of Ethnobiology. 2017;37(3):522 – 541.

4. Liu H, Lee SH, Saunders C. Development of a machine vision system for weed detection during both of off-season and in-season in broadacre no-tillage cropping lands. American Journal of Agricultural and Biological Sciences. 2014;9(2):174–193.

5. Gadekallu TR, Rajput DS, Reddy M, Lakshmanna K, Bhattacharya S, Singh S, et al. A novel PCA–whale optimization-based deep neural network model for classification of tomato plant diseases using GPU. Journal of Real-Time Image Processing. 2021;18:1383–1396.

6. Sabzi S, Abbaspour-Gilandeh Y, Garc´ıa-Mateos G. A fast and accurate expert system for weed identification in potato crops using metaheuristic algorithms. Computers in Industry. 2018;98:80 – 89.

7. DeVries T, Taylor GW. Improved Regularization of Convolutional Neural Networks with Cutout; arXiv:1708.04552 [Eprint]. 2017. Available from: <https://arxiv.org/abs/1708.04552>.

8. Cui Y, Jia M, Lin T, Song Y, Belongie SJ. Class-Balanced Loss Based on Effective Number of Samples; arXiv:1901.05555 [Eprint]. 2019. Available from: <http://arxiv.org/abs/1901.05555>.

9. He H, Garcia EA. Learning from Imbalanced Data. IEEE Transactions on Knowledge and Data Engineering. 2009;21(9):1263–1284.

10. Ghiasi G, Lin T, Le QV. DropBlock: A regularization method for convolutional networks; arXiv:1810.12890 [Eprint]. 2018. Available from: <http://arxiv.org/abs/1810.12890>.

11. Lecun Y, Bottou L, Bengio Y, Haffner P. Gradient-based learning applied to document recognition. Proceedings of the IEEE. 1998;86(11):2278–2324.

12. Cha YJ, Choi W, Buyukozturk O. Deep Learning-Based Crack Damage Detection Using Convolutional Neural Networks. Computer-Aided Civil and Infrastructure Engineering. 2017;32:361–378.

13. Chaki J, Parekh R, Bhattacharya S. Plant leaf recognition using texture and shape features with neural classifiers. Pattern Recognition Letters. 2015;58(Complete):61–68.

14. D AK, Pandi P. A novel wrapping curvelet transformation based angular texture pattern(wctatp) extraction method for weed identification. ICTACT Journal on Image and Video Processing. 2016;06:1192–1206.

15. Hu J, Shen L, Albanie S, Sun G, Wu E. Squeeze-and-Excitation Networks. IEEE Transactions on Pattern Analysis and Machine Intelligence. 2020;42(8):2011–2023.

16. Woo S, Park J, Lee JY, Kweon IS. CBAM: Convolutional Block Attention Module. In: Proceedings of the European Conference on Computer Vision (ECCV); 2018. p. 3–19.

17. Fu J, Liu J, Tian H, Fang Z, Lu H. Dual Attention Network for Scene Segmentation; arXiv:1809.02983 [Eprint]. 2018 [cited 2018 Oct 5]. Available from: http://arxiv.org/abs/1809.02983.

18. Zhao B, Wu X, Feng J, Peng Q, Yan S. Diversified Visual Attention Networks for Fine-Grained Object Classification. IEEE Transactions on Multimedia. 2017;19(6):1245–1256.

19. Evaluation of an autoencoder as a feature extraction tool for near-infrared spectroscopic discriminant analysis. Food Chemistry. 2020;331:127332.

20. Kurmi Y, Chaurasia V. Classification of Magnetic Resonance Images for Brain Tumor Detection. IET Image Processing. 2020;14:1–13.

21. Zheng H, Lin F, Feng X, Chen Y. A Hybrid Deep Learning Model With Attention-Based Conv-LSTM Networks for Short-Term Traffic Flow Prediction. IEEE Transactions on Intelligent Transportation Systems. 2020; p. 1–11.

22. Ma X, Wang H, Geng J. Spectral–Spatial Classification of Hyperspectral Image Based on Deep Auto-Encoder. IEEE Journal of Selected Topics in Applied Earth Observations and Remote Sensing. 2016;9:1–13.

23. Ronneberger O, Fischer P, Brox T. U-Net: Convolutional Networks for Biomedical Image Segmentation. In: Navab N, Hornegger J, Wells WM, Frangi AF, editors. Medical Image Computing and Computer-Assisted Intervention – MICCAI 2015. Cham: Springer International Publishing; 2015. p. 234–241.

24. Kong T, Yao A, Chen Y, Sun F. HyperNet: Towards Accurate Region Proposal Generation and Joint Object Detection. In: 2016 IEEE Conference on Computer Vision and Pattern Recognition (CVPR); 2016. p. 845–853.

25. Gadekallu T, Alazab M, Kaluri R, Reddy P, Bhattacharya S, Lakshman K, et al. Hand gesture classification using a novel CNN-crow search algorithm. Complex & Intelligent Systems. 2021;7:1855–1868.

26. Vasan D, Alazab M, Wassan S, Safaei B, Zheng Q. Image-Based Malware Classification using Ensemble of CNN Architectures (IMCEC). Computers & Security. 2020;92:101748.

27. Bhattacharya S, Reddy Maddikunta PK, Pham QV, Gadekallu TR, Krishnan S SR, Chowdhary CL, et al. Deep learning and medical image processing for coronavirus (COVID-19) pandemic: A survey. Sustainable Cities and Society. 2021;65:102589.

28. Khan S, Tufail M, Khan M, Ahmad Z, Iqbal J, Alam M. A novel semi-supervised framework for UAV based crop/weed classification. PLOS ONE. 2021;16:e0251008.

29. Yanlei Xu BZYJSKYZZG Yuting Zhai. Weed Recognition for Depthwise Separable Network Based on Transfer Learning. Intelligent Automation & Soft Computing. 2021;27(3):669–682.

30. Chollet F. Xception: Deep Learning with Depthwise Separable Convolutions; arXiv:1610.02357 [Eprint]. 2017. Available from: <https://arxiv.org/abs/1610.02357v3>

31. Trong V, GwangHyun Y, JinYoung K, Bao P. Yielding Multi-Fold Training Strategy for Image Classification of Imbalanced Weeds. Applied Sciences. 2021;11:3331.

32. Howard AG, Zhu M, Chen B, Kalenichenko D, Wang W, Weyand T, et al.. MobileNets: Efficient Convolutional Neural Networks for Mobile Vision Applications; arXiv:1704.04861 [Eprint]. 2017. Available from: <https://arxiv.org/abs/1704.04861>.

33. Yan X, Deng X, Jin J. Classification of weed species in the paddy field with DCNN-Learned features. 2020 IEEE 5th Information Technology and Mechatronics Engineering Conference (ITOEC). 2020; p. 336–340.

34. Hu K, Coleman G, Zeng S, Wang Z, Walsh M. Graph weeds net: A graph-based deep learning method for weed recognition. Computers and Electronics in Agriculture. 2020;174:105520.

35. Hoang Trong V, Gwang-hyun Y, Thanh Vu D, Jin-young K. Late fusion of multimodal deep neural networks for weeds classification. Computers and Electronics in Agriculture. 2020;175:105506.

36. Farooq A, Hu J, Jia X. Analysis of Spectral Bands and Spatial Resolutions for Weed Classification Via Deep Convolutional Neural Network. IEEE Geoscience and Remote Sensing Letters. 2019;16(2):183–187.

37. Olsen A, Konovalov DA, Philippa B, Ridd P, Wood JC, Johns J, et al. DeepWeeds A Multiclass Weed Species Image Dataset for Deep Learning. Scientific Reports. 2019;9(1).

38. He K, Zhang X, Ren S, Sun J. Deep Residual Learning for Image Recognition. In: 2016 IEEE Conference on Computer Vision and Pattern Recognition (CVPR); 2016. p. 770–778.

39. Dyrmann M, Karstoft H, Midtiby HS. Plant species classification using deep convolutional neural network. Biosystems Engineering. 2016;151:72–80.

40. Zhang S, Guo J, Wang Z. Combing K-means Clustering and Local Weighted Maximum Discriminant Projections for Weed Species Recognition. Frontiers in Computer Science. 2019;1:4. 41. Bakhshipour A, Jafari A. Evaluation of support vector machine and artificial neural networks in weed detection using shape features. Computers and Electronics in Agriculture. 2018;145:153–160. 42. Hall D, McCool C, Dayoub F, Sunderhauf N, Upcroft B. Evaluation of Features for Leaf Classification in Challenging Conditions. In: 2015 IEEE Winter Conference on Applications of Computer Vision; 2015. p. 797–804.

43. Pereira LAM, Nakamura RYM, de Souza GFS, Martins D, Papa JP. Aquatic weed automatic classification using machine learning techniques. Computers and Electronics in Agriculture. 2012;87:56–63.

44. Ishak AJ, Hussain A, Mustafa MM. Weed image classification using Gabor wavelet and gradient field distribution. Computers and Electronics in Agriculture. 2009;66(1):53–61.

45. Zhou B, Khosla A, Lapedriza A, Oliva A, Torralba A. Learning Deep Features for Discriminative Localization. In: 2016 IEEE Conference on Computer Vision and Pattern Recognition (CVPR); 2016. p. 2921–2929.

46. Zhao J, Liu J, Fan D, Cao Y, Yang J, Cheng M. EGNet: Edge Guidance Network for Salient Object Detection. In: 2019 IEEE/CVF International Conference on Computer Vision (ICCV); 2019. p. 8778–8787.

47. Redmon J, Farhadi A. YOLOv3: An Incremental Improvement; 2018. Available from: <http://arxiv.org/abs/1804.02767>.

48. Szegedy C, Liu W, Jia Y, Sermanet P, Reed S, Anguelov D, et al. Going deeper with convolutions. In: 2015 IEEE Conference on Computer Vision and Pattern Recognition (CVPR); 2015. p. 1–9.

49. Goodfellow IJ, Pouget-Abadie J, Mirza M, Xu B, Warde-Farley D, Ozair S, et al.. Generative Adversarial Networks; arXiv:1406.2261 [Eprint]. 2014. Available from: <https://arxiv.org/abs/1406.2661>.

50. Kingma DP, Ba J. Adam: A Method for Stochastic Optimization; 2017. Available from: <https://arxiv.org/abs/1412.6980v3>.

51. Simonyan K, Zisserman A. Very Deep Convolutional Networks for Large-Scale Image Recognition; arXiv:1409.1556[Eprint]. 2015. Available from: <https://arxiv.org/abs/1409.1556v4>.

52. Tan M, Le QV. EfficientNet: Rethinking Model Scaling for Convolutional Neural Networks; arXiv:1905.11946 [Eprint]. 2019 [cited 2019 Jun 3]. Available from: http://arxiv.org/abs/1905.11946.

**Comments from Reviewer: 1**

1. The reviewer’s comment:

Have the authors made all data underlying the findings in their manuscript fully available?

The authors’ answer:

Thank you very much for the reviewer's suggestion.

To ensure that the data is available to the public, I have uploaded the data to kaggle. If this paper is lucky to be accepted, we will open the data later. The following is the address and the screenshot of the data set.

<https://www.kaggle.com/twisdu/weed-for-deep-learning>

2. The reviewer’s comment:

Is the manuscript presented in an intelligible fashion and written in standard English?

The authors’ answer:

Thank you very much for the reviewer's suggestion. We have carefully corrected the grammar and vocabulary problems of this paper, and standardized the method of writing in English, including the following points: 1. Capitalization of proper nouns, 2. Singular and plural forms of nouns, and 3. Adjustment and rewriting of sentence order. For each specific correction, we mark it in yellow shadow in this paper.

3. The reviewer’s comment:

Last section Conclusion and feature work should be =>Conclusion and Future work.

The authors’ answer:

Thank you very much for the reviewer's suggestion. All of the above issues are addressed in this paper.

4. The reviewer’s comment:

Reference should be provided with each existing method in results Tables.

The authors’ answer:

Thank you very much for the reviewer's suggestion. We have added some citations to the table according to your requirements, and the main citations added are several excellent algorithms proposed by others.
